# 

**Published:** 1897-01

**Authors:** 


### INDEX

TO THE

### HOMŒOPATHIC PHYSICIAN.

### VOLUME XVII, 1897.

PAGE

Adulteration of Food.......... .....385, 430, 431
Albuminuria as Bearing on Puerperal Eclampsia. E. V. Ross.............. 419
Allan, Dr. Arthur G............................... 143
Allen, J. H. The Physician's Highest and Only Calling is to Heal the Sick........................................ 160
Allen, J. Henry. The Molecular *vs.* the Dynamic Theory of Life...... 254
Allopathy, Homœopathy *vs.* Geo. J. Augur........................................ 38
Allopathy, Homœopathy or................ 129
*American Antiquarian and Oriental Journal*.......................................... 72
[illegible]rican Institute of Homœopathy. Jos. T. Cook.............................. 133
[illegible]ican Institute of Homœopathy. E. H. Porter.............................. 136
[illegible]ican Institute of Homœopathy. De Witt G. Wilcox...................... 184
[illegible]erican Institute of Homœopathy: Pharmacopœa of......... ........... 327
[illegible]merican Medical Publishers' Association, Meeting of........................ 143
*American X-Ray Journal*..... ........... 334
Ammonium carbonicum in Scarlet Fever.............................................. 472
[illegible]r. Johann Michael Robert. C. Price.............................. 205
[illegible]rs Sons, Dr.: Vaccination of. M. R. Leverson....... ................ 324
*Annual, The International Medical*, for 1897..................... ........71, 140
Antimonium-tart. in Whooping Cough. 471
*Antiquarian, American and Oriental Journal*......................................... 72
Anti-toxine........................................ 375
Anti-toxine for Diphtheria.................. 325
Antitoxin, Death from....................142, 467
Apology.......................................... 75, 474
Apostrophe to Jenner......................... 467
Appendicitis See "A Matter of Preference"........................................ 110
Appendicitis. Wm. Pepper................. 374
Aqua Marina. Thos. Skinner............. 302
*Arena, The*.......................................... 71
Artistic Spirit in Medicine, The. Stuart Close........................................ 9
Association, Western New York.......... 465
Astigmatism, Ophthalmia and. Levi Hoopes............................................. 513
At War with the Doctors...................... 286
Augur, Geo. J. Homœopathy *vs.* Allopathy........................................ 38
Autoscopy of the Larynx and Trachea. By Alfred Kirstein....................... 72

Back, Repertory of. E. H. Wilsey. 407, 442, 481
Berridge, E. W. Sulphur in Typhoid.. 102
Baylies, B. L. B. Differentiation of Pulsatilla and Phosphorus.......... 360
Bed Feels Hard, The. H. C. Morrow... 494
Bed, Indications Derived from........... 502

PAGE

Beer Bottles, Use of for Samples of Urine 32
Bell, James B. Homœopathic Therapeutics of Diarrhœa.................... 236
Bœnninghausen, Error in Allen s. A. McNeil............. ......................... 133
Boname, L. C. Study and Practice of French in School.......................... 332
Book Notices, 33, 71, 137, 236, 284, 327, 376, 468, 514
Boyle, Charles C. Therapeutics of the Eye.............................................. 73
Bradford, T. L. Pioneers of Homœopathy............................................ 514
Brady, Dr. Edward F....... ............... 74
Brewster, Dr. A. Judson. In Memoriam 132, 229
Brooklyn Hahnemannian Union......... 102
Brooks, Oscar MacDonald, Hahnemann........................................... 74
Bubonic Plague ................................ 57
Bugaboo of Small-Pox. M. R Leverson.............................................. 108
Bugaboo of Small-Pox: Dr. Leverson and The. R. N. Taylor.............. 183
Burnett, J. Compton. Organ Diseases of Women...................................... 284

Calcarea-carbonica. Editorial............ 145, 239, 287, 335, 383
Calcarea and the Proteins. M. R. Leverson.............................................. 283
Calcarea and the Proteins. See "Food and Medicine"............................ 382
Carleton, Edmund. N. Y. Hom. Union 67
Chicago College of Physicians and Surgeons.......................................... 144
Choate, Rufus. Homœopathic Materia Medica............. ........................ 120
Chopchenee in Syphilis...................... 471
Clarke, John H. Heart Repertory...... 237
Close, Stuart. The Artistic Spirit in Medicine.......................................... 9
Symptoms: A Study..................... 85
Colorado Hom. Med. Society.............. 238
Comparison, A..................................... 406
Conference on Materia Medica............ 106
Conium. Editorial................................ 1, 35
Consumptives. Principles for Selection of, for Better Environment.. 137
Contradictions of a Vaccinationist. E. C. Townsend.............................. 372
Convulsions Reflex. E. Vondergoltz. 228
Cook, Jos. T. American Institute of Homœopathy.................................. 133
Corrections. M. R. Leverson.. ........... 441
Cough, with Gaping .......................... 177
Curious Variety of Grippe. Fred. Preston................................................. 56
Curryer, Wm. F. Dunham Medical College............................................ 373
Cyclopedia of Drug Pathogenesy, Repertory to. R. Hughes........... 379

Death from Vaccination....................... 471

PAGE
Denver Hom. Med. College and Hospital... 144
Des Moines Homœopathic Medical Society... 463
Dewey, W. A. Materia Medica Conference... 106
Diagnosis, The Eye as an Aid in. E. H. Linnell... 377
Diarrhœa. Hom. Therapeutics of. Jas. B Bell... 236
Differentiation of Pulsatilla and Phosphorus, with Illustrative Observations. B. L. B. Baylies... 360
Digitalis in Pneumonic Fever. E. V. Ross... 177
Diphtheria, Anti-toxine for... 325
Diphtheria, March of, in the Southwest of France... 31
Disease and Drug Action. W. S. Talcott... 459
Drake, Olin M. Necrosis of Superior Maxillary... 451
Drunkenness, Inoculation for... 473
Dunham Medical College. W. F. Curryer... 373
Dynamic Theory of Life, The Molecular *vs.* J. H. Allen... 254
Dysentery. P. P. Wells... 503
Dysuria. L. M. Stanton... 277

Editorials......1, 35, 75, 111, 145, 187, 239, 241, 287, 335, 383, 433, 473
Eggleston. Principles of Medicine... 332
Electricity in Electro-therapeutics. E. J. Houston... 109
Electro-therapeutist, The... 381
Eradication of Mongrelism. C. F. Menninger... 339
Error in Allen's Bœnninghausen. A. McNeil... 133
Eye as an Aid in General Diagnosis. By E. H. Linnell... 377
Eye-sight Testing... 382
Eye Strain in Health and Disease. By A. L. Ranney... 329
Eye, Therapeutics of. By Charles C. Boyle... 73

Faulkner, Dr. M. R... 382
Fincke, B. For High Potencies... 44
Provings of the X-Rays...304, 306
Dr. Talcott's Paper... 461
Flora, Study of the American Medicinal... 279
Food, Adulteration of...385, 430, 431
For High Potencies. B. Fincke... 44
French, Study and Practice of... 332
Fun for Doctors.....74, 110, 142, 186, 382, 472
Funk & Wagnalls Company... 34

Gaping, with Cough... 177
Geddes, Dr. Annie Lowe—Removal... 471
Germ Theory, The... 232
Gliddon, Jean Ida Mackay... 382
Grippe, A Curious Variety of. Frederic Preston... 56
*Gross' Comparative Materia Medica*. 331

Hahnemann. Oscar MacD. Brooks... 74
Hahnemann Association of New York.. 74
Hahnemann Medical Association of Iowa... 143
Hahnemann Medical College, Philadelphia... 143
Hahnemann Monument... 466
Has a Moon on His Neck... 142

PAGE
Hasbrouck, Dr. Jos... 382
Heart Repertory. By John H. Clarke. 237
Hering on Typhoid Fever. Editorial. 187, 289
Heysinger, I. W. Scientific Basis of Medicine... 468
His Kind of a Path... 74
Holmes, Dr. H. P... 285
Holmes, Horace P. Palladium... 27
Homœopathic Materia Medica. Rufus Choate... 120
Homœopathic Medical Society of Tennessee... 143
Homœopaths to Receive Recognition... 109
Homœopathy *vs.* Allopathy. Geo. J. Augur... 38
Homœopathy or Allopathy... 129
Homœopathy, The Law of Cure and the Basis of a True Psychology. J. W. Thomson... 265
Homœopathy, Missouri Institute of... 74
Homœopathy: Not Enough Taught. J. M. Selfridge... 369
Homœopathy, Pioneers of. Review of. 514
Homœopathy, Veterinary. By J. S. Hurndall... 138
Hoopes, Levi. Ophthalmia and Astigmatism... 513
Horse: Veterinary Homœopathy of. Review... 138
Houston, E. J. Electricity in Electro-therapeutics... 109
Hughes, Richard. Repertory to the Cyclopædia of Drug Pathogenesy 379
Hurndall, J. S. Veterinary Homœopathy of the Horse... 138
Hydrocyanic Acid Case, A. A. S. Ironside... 449

Infection, The Time of. J. M. Selfridge... 424
In Memoriam. Dr. A. Judson Brewster... 132
Samuel Hahnemann Jackson... 131
Dr. Stephen H. Seward... 100
Drs. S. A. Seward and A. J. Brewster 229
Dr. Mary Bates Stevens... 176
Inoculation for Drunkenness... 473
International Hahnemannian Association...75, 111, 172, 186, 238, 272, 471
International Hahnemannian Association, Members of...75, 111
*International Journal of Surgery*... 286
*International Medical Annual*, for 1897... 71, 140
Investigation of Infantile Scurvy... 374
Ironside, A. S. A Hydrocyanic Acid Case... 449

Jackson, Samuel Hahnemann. In Memoriam... 131
James, Dr. Sanitarium... 471
James, Walter M. Conium...1, 35
Calcarea-carb...145, 239 287, 335, 383
Hering on Typhoid Fever... 187
Inoculation for Drunkenness... 473
Matter of Choice, A... 474
Jenner. Apostrophe to... 467

*Keil's Medical, Pharmaceutical and Dental Directory*... 381
King, William Harvey. Treatise on Spermatorrhœa, Impotence and Sterility... 378
Kirstein, Alfred, Autoscopy of Larynx and Trachea... 72

PAGE
Larynx and Trachea, Autoscopy of..... 72
Ledyard, W. E. *Organon* and Materia Medica Club of California......... 230, 320, 321
Leprosy and the Charity of the Church. L. W. Mulhane.......................... 333
Leverson, Dr., and the Bugaboo of Small-Pox. R. N. Taylor........... 183
Leverson, M. R. Calcarea and the Proteins.................................... 283
Corrections................................ 441
Dr. McNeil on Vaccination.......... 452
Positive *vs.* Negative Facts........... 423
Vaccination of Dr. Amthor's Sons. 324
Vaccination: The Royal Com...... 132
Vaccination in the Light of the Royal British Commission...3, 76, 112, 147, 206, 242, 290, 347, 391, 434
Vaccination Testimony, The....... 226
Leverson on Vaccination. J. W. Thomson.................................... 323
Linnell, E. H. The Eye as an Aid in General Diagnosis...... ............. 377
Lippe's Lectures, Notes on.................. 433
Lutze, Dr. F. H.................................. 186

Macfarlan, M. The Power of Potentizing.......................................... 173
Malaria in Russia.............................. 465
Manning, Guy E. *Organon* and Materia Medica Club.......... . ...... 424
Marasmus—Opium. E. V. Ross.......... 97
March of Diphtheria in the Southwest of France........................ .............. 31
Married ........................................... 33
Martin, G. H. *The Organon* and Materia Medica Club of California..... 429
Materia Medica Conference................. 106
Materia Medica, Editorials on............ 111
Materia Medica, Gross's Comparative.. 331
Materia Medica, Homœopathic. Rufus Choate.......................................... 120
Matter of Choice, A.... ..................... 474
Matter of Preference, A...................... 110
Maxillary, Superior, Necrosis of........ 451
Medical Supply Companies................ 185
Medicine, The Artistic Spirit in. Stuart Close ....................................... 9
Medicine, Principles of. By E. R. Eggleston ........................................ 332
Menninger, C. F. Eradication of Mongrelism......................................... 339
McNeil, A. An Error in Allen's Bœnninghausen.................................... 133
Vaccination................................. 367
McNeil on Vaccination. M. R. Leverson ................................................ 452
Middletown Homœopathic Hospital... 238
Missouri Institute of Homœopathy..... 74
Missouri State Medical Association, 144, 238
Molecular *vs.* the Dynamic Theory of Life. J. H. Allen.......................... 254
Mongrelism, Eradication of. C. F. Menninger ...................................... 339
Monograph of Diseases of Nose and Throat. G. H Quay...................... 376
Moon on His Neck, Has a.................... 142
Morphine Sulphate............................ 100
Morphine, Symptoms from. W. H. Phillips........................................ 99
Morrow, H. C. The Bed Feels Hard... 494
Mulhane, L. W. Leprosy and the Charity of the Church................ 333

Necrosis of the Superior Maxillary. O. M. Drake...................................... 451
Nephritis, Acute, Case of. Ella M. Tuttle......... .................... ............ 421
New York Homœopathic Union, The. 20, 67, 233, 274, 453, 475
Nitric-acid for Itching of Nose. J. W. Thomson...... ............................ 30
Northern Indiana Association............ 181
*Northwestern Monthly*......................... 237
Nose, Itching of, Nitric Acid for. J. W. Thomson.................................... 30
Not Enough Homœopathy Taught. J. M. Selfridge ............................ 369
Nose and Throat, Monograph of Diseases of. G. H. Quay................... 376
Notes and Notices, 74, 109, 142, 186, 238, 285, 382, 432, 471
Notice ............................................... 111
Nux-moschata, Verification of. J. C. White................................................ 326

*Observer, The*........................................ 33
Obstetrical Surgery and Gynæcology... 144
Open Court............................................ 285
Ophthalmia and Astigmatism. Levi Hoopes ................................................ 513
Opinions on Vaccination...................... 301
Opium in Marasmus. E. V. Ross...... .. 97
Organ Diseases of Women. J. C. Burnett............................................... 284
*Organon* and Materia Medica Club of the Bay Cities of California. 60, 63, 65, 103, 125, 126, 178, 230, 320, 321, 424, 429

Palladium. Horace P. Holmes....... ... 27
Path, His Kind of a.............................. 74
Peculiarities of the X-Rays.................. 373
Pennsylvania, Homœopathic Medical Society of............................................ 238
Pepper, Wm. Appendicitis................. 374
Pharmacopœa of the American Institute of Homœopathy...................... 327
Phillips, Dr. A. W.................................. 238
Phillips, W. H. Symptoms from Morphine ................................................ 99
Phosphorus, Pulsatilla and, Differentiation of. B. L. B. Baylies ...... 360
Physicians' Highest and Only Calling is to Heal the Sick. J. H. Allen. 160
Physicians' Visiting List for 1897......... 33
Pioneers of Homœopathy. By T. L. Bradford........................................ 514
Pneumonic Fever, Digitalis in. E. V. Ross.............................................. 177
Porter, Eugene H. American Institute of Homœopathy......... ........ 136
Positive *vs.* Negative Facts. M. R. Leverson ........................................ 423
Potencies, For High. B. Fincke......... 44
Potentizing, Power of. M. Macfarlan. 173
Power of Potenizing. M. Macfarlan... 173
Preston, Frederic. A Curious Variety of Grippe............................................ 56
Price, Elias C. Dr. Johann Michael Robert Amthor .... ........................ 205
Principles for Selection of Consumptives for Better Environment..... 137
Proving of Terebinthina. E. V. Ross. 227
Provings of the X-Rays. B. Fincke, 304, 306
Psychology, Homœopathy the Basis of a True. J. W. Thomson............. 265
Puerperal Eclampsia, Albuminuria as Bearing on. E. V. Ross............. 419
Pulsatilla and Phosphorus, Differentiation of. B. L. B. Baylies............ 360
Pyrogen. W. A. Yingling.................... 106

PAGE

Quay, Geo. H. Monograph of Diseases of Nose and Throat...... 376

Ranney, A. L. Eye Strain in Health and Disease...... 329
Reed, Dr. W. L...... 238
Repertory of the Back. E. H. Wilsey......407, 442, 481
Repertory to the Cyclopædia of Drug Pathogenesy. By Richard Hughes...... 379
Rœntgen Rays, Provings of. B. Fincke......304, 306
Ross, E. V. Albuminuria as Bearing on Puerperal Eclampsia...... 419
Digitalis in Pneumonic Fever...... 177
Marasmus—Opium...... 97
Proving of Terebinthina...... 227

Scarlet Fever, Ammon-carb. in...... 472
Scientific Basis of Medicine. By I. W. Heysinger...... 468
Scott, Dr. Catharine V. C...... 144
Scurvy, Infantile, Investigation of...... 374
Seashore Residence Advisable for Patients with Nervous Troubles...... 143
Selfridge, J. M. Not Enough Homœopathy Taught...... 369
The Time of Infection...... 424
Vital Force...... 188
Seward, Dr. S. A. In Memoriam...100, 229
Skinner, Thomas. Aqua Marina...... 302
Small-Pox, Bugaboo of. M. R. Leverson...... 108
Society of Homœopathicians......144, 186
Southwestern Homœopathic Medical College...... 142
Spermatorrhœa, Treatise on. W. H. King...... 378
Stamford Hall......33, 109, 432
Stanton, Laurence M. Dysuria...... 277
Sterility and its Rational Treatment. E. Vondergoltz...... 163
Stephens, Governor...... 285
Stevens, Dr. Mary Bates. In Memoriam...... 176
Storer, Dr. John. A Card...... 33
Storer, John. In Memoriam: Samuel Hahnemann Jackson...... 131
Study of the American Medicinal Flora 279
Study and Practice of French in School. L. C. Boname...... 332
Sulphur in Typhoid. E. W. Berridge.. 102
Symptoms from Morphine. W. H. Phillips...... 99
Symptoms: A Study. Stuart Close.... 85
Syphilis, Chopchenee in...... 471

Taking the Case. W. A. Yingling...... 385
Talcott, W. S. Disease and Drug Action...... 459
Talcott's Paper, Dr. B. Fincke...... 461
Taylor, Robert N. Dr. Leverson and the Bugaboo of Small-Pox...... 183
Tennessee, Hom. Medical Society of... 143
Terebinthina, Provings of. E. V. Ross.. 227
Therapeutics of the Eye. By Chas. C. Boyle...... 73
Thomson, J. W. Homœopathy, the Law of Cure and the Basis of a True Psychology...... 265
Leverson on Vaccination...... 323
Nitric-acid for Itching of Nose...... 30
Townsend, E. C. Contradictions of a Vaccinationist...... 372

PAGE

Treat, E. B. & Co...... 286
Treatise on Spermatorrhœa, Impotence, and Sterility. By W. H. King...... 378
Tussey, Edgar A. Principles for Selection of Consumptives, etc...... 137
Tuttle, Ella M. A Case of Acute Nephritis...... 421
Typhoid Fever, Hering on...... 187, 289
Typhoid, Sulphur in. E. W. Berridge. 102

Underwood, Dr. Maro F......33, 186
Union, Brooklyn Hahnemannian...... 102
Union, New York Homœopathic...... 20, 67, 233, 274, 453, 475
United States Department of Agriculture: Adulteration of Food...430, 431
Urine, Use of Beer Bottles for Samples, 32

Vaccination, Death from...... 471
Vaccination of Dr. Amthor's Sons. M. R. Leverson...... 324
Vaccination, Dr. McNeil on. M. R. Leverson...... 452
Vaccination. Editorial...... 433
Vaccination. A. McNeil...... 367
Vaccination, Leverson on. J. W. Thomson...... 323
Vaccination in the Light of the Royal British Commission. M. R. Leverson...3, 76, 112, 132, 147, 206, 242, 290, 347, 391, 434
Vaccination in the Light of the Royal British Commission. A. Wheeler. 371
Vaccination Lies...... 375
Vaccination, Opinions on...... 301
Vaccination: The Royal Commission. M. R. Leverson...... 132
Vaccination Testimony. M. R. Leverson...... 226
Vaccinationist. Contradictions of a. E. C. Townsend...... 372
Verification of Nux-moschata. J. C. White...... 326
Veterinary Homœopathy in its Application to the Horse. By J. S. Hurndall...... 138
Vital Force. J. M. Selfridge...... 188
Vital Force. Editorial...... 241
Vondergoltz, Eric. Reflex Convulsions...... 228
Sterility and its Rational Treatment...... 163

War with the Doctors...... 286
Wells, P. P. Dysentery...... 503
Western New York Association...... 465
Wheeler, A. Vaccination in the Light of the Royal British Commission...... 371
White, J. C. Verification of Nux-moschata...... 326
Whooping Cough, Antimon-tart. in..... 471
Wilcox, De Witt G. The American Institute of Homœopathy...... 184
Wilcox, Emma D. New York Homœopathic Union......233, 453
Wilsey, E. H. Repertory of the Back. 407, 442, 481

*X-Ray Journal*, American...... 334
X-Rays, Peculiarities of...... 373
X-Rays, Provings of. B. Fincke... 304, 306

Yingling, W. A. Pyrogen...... 106
Taking the Case...... 385

VOL. XVII. JANUARY, 1897. No. 1.

### THE HOMŒOPATHIC PHYSICIAN,

A Monthly Journal of Homœopathic Materia Medica and Clinical Medicine.

*" He is a freeman whom truth makes free, and all are slaves beside."*

*" Seek the Truth: come whence it may, cost what it will."*

EDITED AND PUBLISHED BY

WALTER M. JAMES, M. D.

NOTICE.

***All articles for publication, books for review, business communications, etc., must be sent to Editor, 1231 Locust Street, Philadelphia.***

***Subscribers will please notice that this Journal will be sent until arrears are paid and it is ordered discontinued.***

